# Techno-Cultural Factors Affecting Policy Decision-Making: A Social Network Analysis of South Korea’s Local Spatial Planning Policy

**DOI:** 10.3390/ijerph17238746

**Published:** 2020-11-25

**Authors:** Eun Soo Park, An Yong Lee

**Affiliations:** 1 Department of Architecture, Sahmyook University, Seoul 01795, Korea; espark@syu.ac.kr; 2 Department of Smart Factory, Korea Polytechnics, Incheon 21417, Korea

**Keywords:** local spatial planning stage, policy decision-making, social network analysis, convergence analysis, text mining analysis

## Abstract

Increasing interest in various local construction forms necessitate examining its link to human life. Construction culture should be adapted and applied to the contemporary context to create a harmonious coexistence with diverse local cultures and to strengthen regional sustainability, avoiding the rigid, one-dimensional local construction development. Thus, this study aims to analyze the factors of influence needed for policy decision-making at the local spatial planning stage, with regional technologies and cultural contents from a convergent perspective taken into consideration. This study derived tangible and intangible policy decision-making factors during the spatial planning stage using text mining analysis. Additionally, social network analysis was also used to seek multi-angle correlations among factors. Through big data analytics, 16 key decision-making contents in the spatial planning stage were derived, with ‘regional development, urban policy’ as most influential. Such a result indicates the need for regional and urban policy engagement with strategic development from a holistic perspective—in view of socio-cultural relations and forms of change—and local perceptions of spatial value and significance affecting decision-making in the local spatial planning stage (LSPS). Understanding the decision-making process in the spatial planning stage requires a holistic approach with both visible technological factors (structure, form, and construction method) and invisible cultural factors (ways of life projected during space formation, zeitgeist, religion, learning, and art) included.

## 1. Introduction

Along with rapid changes in the Information Age, the culture of each region representing a given era is becoming even more segmented and complex. Increasing cultural diversity gradually turns regional characteristics, factors, and influence, (for example, invisible factors, such as local spirit, ideas, customs and traditions, and regional characteristics of economic, policy, geography, and environmental factors), which used to be understood from a fragmentary perspective in the past, more difficult in terms of any single field. Likewise, predicting and responding to future changes is also becoming even more challenging. Advances in information technology for knowledge delivery have led to the creation or destruction of diverse cultural content forms, many of which cannot be explained from a single viewpoint.

Therefore, the regional development sector also needs to consider the diversity of local culture in the establishment of local construction policies by adopting a broader perspective and approach to the preservation, development, and management of historical or cultural assets, including natural resources, unique to each region. Currently, Government policies in place on regional development operate from a technological-based regional development context. According to the current Ministry of Land, Infrastructure and Transport Act, 46 factors for impact checklists are provided for decision-making in the local spatial planning stage. Among these factors, only two cultural factors are considered (the review of the humanities and environment, the inclusion of cultural heritage preservation values). As a cultural and regional development framework, this would be a good model of balanced regional development that can draw on the regional identity and self-esteem of local residents, as well as maximize the use of traditional contents and local traits that are passed on in each region. Accordingly, regional construction may draw out unique innovations and development of diverse cultural contents if policy measures at the government level are taken into consideration from multiple angles in the local spatial planning stage (LSPS).

In particular, although specific details needed to establish a local spatial plan will differ according to the scale and requirements of construction in each region, various tangible and intangible factors generally connected with technological factors relevant to construction, such as the characteristics of each region, direction, and indicators set for the plan, adoption of a spatial structure, conservation and management of the environment, infrastructure, as well as economic, social, and cultural growth exist [1]. Various intangible elements—historical facts, political background, cultural trends, and climate conditions of a given region—pervade that region in different forms as part of the local life and culture.

Julia and Michael [2] have investigated the methods of analyzing the contents of oral traditions, tales, etc., handed down from the local private sector, local participation and observation, study of official organizations, community research designs, and field surveys of humans and their culture.

In 2006, the Korea Rural Development Administration (RDA) [3] conducted research on amenities in urban and rural areas. This study developed the discovery and inventory of local amenity resources, derived important resources and classification systems, and conducted the valuation of resources. Based on this, the factors and characteristics of vitalization of local resources were analyzed to develop regional planning guidelines and technologies and to propose policy proposals and commercialization measures.

For the purposes of this study, region type refers to a special space where particular traditions and cultures have taken shape and are passed on, and the flow of events in accordance with the local form of culture comprise the continuous production of time. In this context, ‘region’ signifies a living space where humans seek to lead their daily lives imbued with happiness [4]. Additionally, humans are members of local communities that produce the cultures adapted to various conditions and environments and subsequently passed them on to create their future. This is similar to Carl Sauer’s theoretical approach. In 1925, Carl Sauer defined a cultural landscape as a natural landscape that had been modified by a cultural group. The cultural landscape will change itself according to the conditions of a given culture and over time, experience development, go through stages or reach almost the last stage of the development cycle [5].

In line with the abovementioned culture flow, construction policies in each region have also coexisted in relation to various factors. In recent regional development, the aesthetic and cultural elements have a great influence on the urban image and cultural industry. It is necessary to define the harmony between buildings and surrounding spaces and the consideration of urban landscape as public elements. It is also necessary to implement construction policies that emphasize the importance of the quality of local development in the environment and culture. Regions thus become points of contact for the symbiosis of the past and present taking place in the space that humans maintain. The present study focuses on identifying such points of contact through cultural factors that can be used to prepare for the future by looking back on the past and reflecting on the present. Technological and cultural factors indigenous to a region and relevant to the establishment of local construction policies are thus analyzed to examine the diverse policy-related local factors for promoting the coexistence of technology and culture relevant to decision-making in LSPS.

Therefore, the purpose of this study is to examine the previously mentioned cultural factors of the local space as well as the technical development conditions previously reflected in the LSPS decision-making process. Additionally, this study analyzes which of the cultural factors analyzed has an effect on the technical factors. Through this, the research can contribute to the creation of regional development as a future-oriented space element into a living space where humans seek to lead their daily lives imbued with happiness, rather than just infrastructure development.

As essential data that shed light on the correlations relevant for decision-making, the study results can be utilized to derive policy decision-making indicators through the application of data analysis methodology and the convergence perspective.

## 2. Literature Review: Construction Planning Stage and Local Spatial Planning Stage

In general, construction planning is a perfunctorily part of the predesign stage and is comprised of tasks such as setting the course of the design and conducting a feasibility study for the construction project. In reality, however, it can be designated as a comprehensive coordination task for the entire design process, from project conceptualization to actual construction work [6]. In particular, the construction planning stage is a very important period that affects the calculation of about 80% of the total project cost. Even small-scale construction projects can cause defects in actual construction if they do not have a clear concept in the planning stage [7]. Therefore, construction planning is not confined to the predesign stage but extends from the employer’s project proposal stage to the stage just before project implementation. Likewise, the planning task comprehends in its scope the preliminary spatial plan, working design, and actual spatial plan [8].

The main task of the planning stage can be the establishment of the basic course of the project at a certain level, similar to the concept establishment process in computer programming [9]. In other words, it may be referred to as the stage for carrying out tasks such as decision-making by the employer or project participants, conducting a feasibility study for the project, specifying the requirements for the target object, and initial space planning [10]. It is also the stage wherein initial project information is generated with the project launch, providing basic information such as the project concept, aim, intended use, and scale. Generally, such information includes site environment, marketability, project-related requirements (scale, shape, space, and others), and initial space plan, which are transformed into a construction model by going through the stages of concept design, working design, actual construction, and maintenance. In a broader sense, the planning stage makes up the design establishment process.

### 2.1. Classification of the Characteristics of the Local Spatial Planning Stage and Decision-Making Targets

Accurate decision-making in the spatial planning stage may vary greatly depending on the application characteristics of the planning stage. Therefore, it is necessary to consider the timing and purpose of the application that reflects the various characteristics of the planning phase [11]. While the spatial planning stage has the same overall analytic framework across various cases, there are differences in the core analytic tasks based on the project’s development domain, promotion method, and type [12]:

- Scale of the project development domain. Relevant regulations established by the Ministry of Land, Infrastructure, and Transport (MOLIT), distinguish domains according to the scale such as metropolitan, city, county, district, and others.
- Project promotion method and developer such as private and government-funded investment.
- Project type (distinguished by work type and function), including size and classification of the social infrastructure.

These variations thus imply that planning stage tasks have different characteristics with regard to applied items and component technologies according to the project’s target object [13]. The spatial planning stage program may be referred to as a process involving a systematic interpretation of the organizational, group, and individual aims and duties derived from various elements such as activity, human, and equipment relationships through relevant programming.

### 2.2. Decision-Making Policy System

As the regions covered in this study are social living spaces, decision-making in the spatial planning stage is understood in terms of valuable spaces where cultural preservation and technological development are harmonized during the promotion of local construction. Although specific details required for decision-making in the LSPS will differ according to the construction scale and requirements, various factors are generally connected with technological factors relevant to construction such as the characteristics of each region, direction, and indicators set for the plan, adoption of a spatial structure, conservation, and management of the environment and infrastructure, as well as the economic, social, and cultural growth.

**Figure 1:**
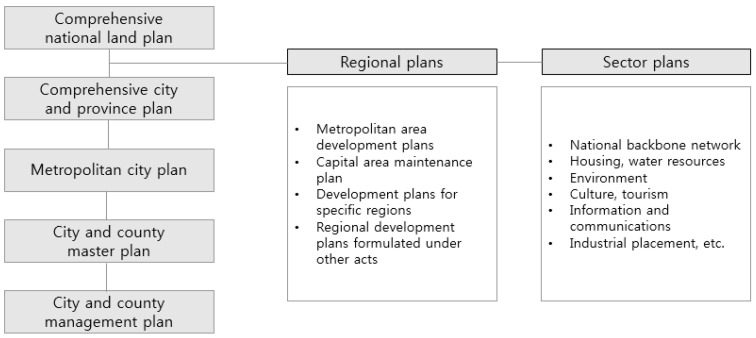
National land space planning system.

In this study, the selected local spatial plans were divided into regional plans and sectoral plans, following the Framework Act on the National Land. Figure 1 shows how the said Act is divided into sublevel plans.

### 2.3. Decision-Making Agents

**Figure 2:**
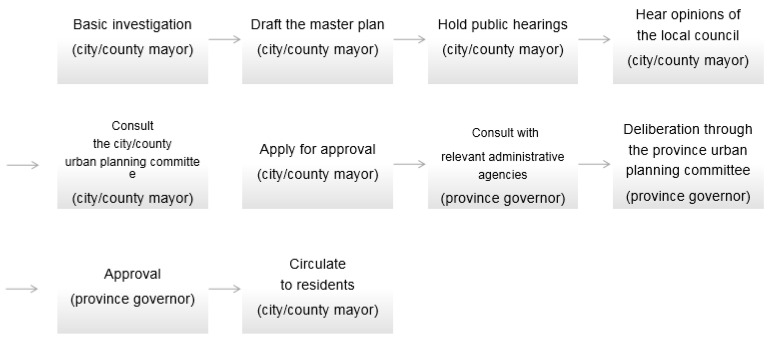
City/county master plan implementation procedure.

According to enforcement procedures for the relevant laws [14], the key policy-makers in the national land space planning system are the heads of local governments, and the feasibility study for a given plan and deliberation thereof are generally conducted through an urban planning committee, which is generally comprised of experts and representatives of residents. Such committees include the Central Urban Planning Committee, which deliberates on large-scale urban development projects, as well as their local counterparts that deliberate on major projects involving local governments in the national land-space planning system [14]. Figure 2 shows how these committees generally implement their master plan.

These committees aim to prevent the rigid operation of urban planning that regulates private property rights for the public’s benefit, as well as ensure flexibility appropriate to local circumstances through the management of deliberation and counseling on proposed urban plans. Recently, however, questions regarding the professionalism and fairness of the local urban planning committee members in their evaluation arose, based on the result of the survey on the operation of the urban planning committee and architecture committee of local governments [15]. The main issues involved are subjective judgments based on qualitative factors, insufficient data on licensing criteria, lack of review and review checklists, and excessive documents that are not relevant. Moreover, the lack of consistency in the deliberation standards employed in each local government has led to confusion in local land use. Additionally, there are no consistent and explicit criteria to adjudicate among the contradictory opinions of different committees. In this study, organizations in charge of the final deliberations in spatial planning, such as the local urban planning committees, are thus selected as target agents.

## 3. Materials and Methods

First, key search terms were selected and analyzed through consultations via interviews and e-mail queries with two practical experts in construction work, two experts in construction management, and two experts in the field of spatial culture from 1 May to 15 May 2020. Research feedback was received through data revision and supplementation. The selected search terms were used to collect data from a total of 1705 research papers in Korean academic journals from 1970 to 2020 [16]. Then, data from Korean MOLIT (Ministry of Land, Infrastructure, and Transport, the Korean government) news articles and KERIS (the Korea Education and Research Information Service) research papers were collected, and the top 50 keywords were derived and subsequently categorized. The Delphi method was used to derive the major factors affecting the research contents, which were subsequently placed in a matrix diagram. The Delphi method of this study is a decision-making methodology that effectively collects expert opinions through several surveys and interviews. This study conducted three rounds of open expert surveys to advance the convergence opinions of technology and cultural elements. The three rounds of Delphi methods have resulted in the final 16 cultural and technological factors being derived through examination, supplementation, and review. Expert opinions on results to derive more objective information on factors influencing decision-making in the LSPS were also collected. NetMiner 4.0 (Cyram, Gyeonggido, South Korea) was then used for the social network analysis of key contents to academic research data. Subsequent sections explain in more detail how the study proceeded.

### 3.1. Text Mining Analysis

Big data refers to the construction of a data system using hardware such as storage media or servers to process huge amounts of data. On the contrary, this study intends to focus on the analysis of stored data to produce valuable data through text mining analysis, which extracts valuable information from texts comprising unstructured data. It is an effective method for structuring data found in various texts and documents.

**Figure 3:**
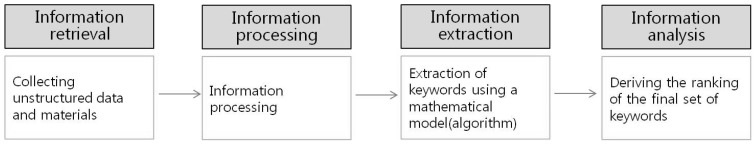
Text mining analysis.

In this study, R programming (release 3.6.3, The R Foundation, Vienna, Austria), a programming language and software environment for statistical computing, was used to extract important information from unstructured texts of academic papers and news articles, as well as analyze related contents [17]. Figure 3 shows the text mining framework adopted in this study.

For keyword derivation, words found in the collected data are first extracted, stop words are removed, and synonyms are then replaced by representative words. Correlations among these words were subsequently analyzed. In this study, the programming language and software suite R (version 3.6.3) was used to collect, organize, and analyze data. KoNLP (Korean Natural Language Process toolkit, Heewon Jeon, Seoul, South Korea) and word cloud packages for R were used for data analysis. Derived techno-cultural factors from these keywords and research data on decision-making in the LSPS were then analyzed.

Keyword extraction used in this study utilized R programming package, and a dictionary provided by KoNLP was used for word extraction. The process of text mining analysis was performed in the order of noun extraction, frequency table data frame creation, limiting word setting, and word-specific extraction. The source code used for text mining is stated in Appendix B.

#### 3.1.1. Collection and Extraction of Data Related to Decision-Making in LSPS

The scope of this study region is a special space for places inherited by the formation of traditions and cultures for humans. This is a continuous result of time made by the flow of events in accordance with the cultural types in the region. Regions mean a place of life where humans want to enjoy happy daily lives. Here, humans are members of the community who create culture according to various conditions and circumstances and inherit it to create the future. Regional development policies have also been co-existing in this cultural trend due to various influences. Therefore, this study focuses on looking back on the past and finding a point of contact as a cultural influence factor that reflects the present.

Search terms selected for this study were ‘construction’, ‘civil works’, ‘national land’, and ‘city’, along with the basic terms ‘culture’ and ‘region’. Table 1 shows how these terns were categorized and the corresponding number of extracted words. These data were collected through the Research Information Sharing Service (RISS), provided by the Korea Education and Research Information Service (KERIS).

Press releases from the Urban Policy Division of the Ministry of Land, Infrastructure, and Transport (MOLIT) were utilized as additional data sources. The division is in charge of all work related to regional national land development. The said press releases provide clear statements of the government policies, indicating the most important issues in local spatial planning in the course of national land development. Therefore, they contain important keywords related to local construction policies and national policy as well as technological, legal, and institutional terminology that can be used as basic data. Accordingly, 144 relevant press releases from the Urban Policy Division posted on the MOLIT website, Government 3.0, from 21 February 2006 to 20 February 2020 were used as a basis for information on factors relevant to decision-making in the spatial planning stage.

The data from this study were collected in two methods. First, information data of academic papers were collected in XML (Extensible Markup Language) format through the API (Application Programming Interface) service provided by RISS. Second, the press release data of the government portal bulletin board of the Ministry of Land, Infrastructure, and Transport (MOLIT) was collected through R programming-based web crawling.

#### 3.1.2. Keyword Derivation and Classification through Text Mining Analysis

Text mining was used to analyze keywords from the collected 1705 research papers and 144 press releases. Using software suite R to extract the synonyms and stop words, at least 30 of the most frequently repeated keywords were derived in each search category (Table 2), thus arriving at a more accurate assessment of the influence of the associated factors.

Among the derived keywords, those expressing the same content, such as those involving the mixed-use of Korean and English or different terms having the same meaning, were merged together. Related keywords were thus grouped first into sets and sorted according to a classification scheme that reflects the opinions of experts, before the analysis [18]. Fifty of the most frequent keywords in the data collected from all target papers and articles were subsequently derived from investigating the cultural and technological factors relevant to decision-making in the LSPS.

Given the nature of text mining analysis, the higher-ranked keywords in Table 2 above comprise the most basic terminology for decision-making in the spatial planning stage. Keywords with a frequency of at least 300, such as culture, space, region, history, and policy, have cultural and technological connotations. In particular, words with cultural meaning—various factors associated with the region to be considered for decision-making in the spatial planning stage—or those that represent social phenomena, such as practical change and diversity, were derived as keywords related to policy considerations in the decision-making of the spatial planning stage.

Based on the 50 derived keywords, the classification of factors was carried out through the preceding study of extant literature and round one of the consultations with experts. Taking into account the diversity of decision-making in the spatial planning stage, the cultural and technological factors were subsequently classified.

For the classification of cultural factors, basic codes forming the cultural basis of decision-making in the spatial planning stage—local ethos, thought and religion—are shaped by the diverse cultural backgrounds and local history of a given region. Notably, these are relevant to the regional development background, urban design, planning, and policy decision-making among others.

Regarding the classification of technological factors, 10 factors relevant to decision-making in the LSPS were identified through consultation with experts, based on this study’s aims and an analysis of the deliberative criteria on technologies given in the Framework Act on the National Land.

The cultural and technological factors relevant to decision-making in the LSPS may be regarded as the sum of mutually opposed concepts, but they can all be regarded as spatial contents created through various interrelationships based on local construction. For example, thought and tradition greatly influenced specific policies and technological advances. Additionally, information is provided to locals as specific systems and policies through a harmonious application of the relevant facilities and technologies. The concept of scenic view as a practical, aesthetic space, which is not found in extant discussions of cultural and technological factors, is also important; therefore, a separate keyword that can reflect this concept was introduced.

### 3.2. Delphi Research Design

The Delphi method is a decision-making technique for generating a consensus among experts through several rounds of the survey. This method is used when there are few materials to refer to, and expert opinions can be used as important information. Thus, it was deemed appropriate to re-examine the results derived in this study which supplement the data. As the current study’s aims lie in analyzing the content, not just in the field of regional policy and construction but in the wide-ranging field of policy decision-making through the inclusion of a sociocultural approach, the Delphi technique would be effective as it allows for a more convergent approach that arrives at a consensus by aggregating diverse opinions from a group [19]. Here, the Delphi technique proceeded through individual meetings and three rounds of open-ended questionnaires and e-mail questionnaires sent to a panel of experts from construction, environment, work, design, and management to analyze relevant technological factors and to experts in spatial culture, spatial philosophy, and regional policy for cultural factors. See Table 3 for the detailed stages of the research design.

#### 3.2.1. Derivation of Factor Indicators through Delphi Analysis

Table 4 shows the indicators derived through the Delphi survey. Here, 16 indicators of technological factors and 31 indicators of cultural factors were derived. The 16 indicators of techno-cultural factors (Table 5) were subsequently examined to derive the convergent factors.

Mainly, the Delphi panel experts examined the validity of the preliminary factor indicators to check whether the research aims and direction of the analysis were adequately reflected in these indicators. Taking the experts’ consensus into account, three Delphi survey rounds were conducted to secure an adequate level of validity for the factor indicators.

#### 3.2.2. Matrix Construction and Weight Assignment: Correlation Analysis

After deriving the techno-cultural factor indicators, a matrix to determine the actual correlations among the factors was constructed. NetMiner 4.0 (Cyram, Gyeonggido, South Korea), a social network analysis program developed by Cyram, was used to analyze the matrix.

In keeping the data format of NetMiner 4.0, the matrix created in this study is comprised of a network or relational dataset that can be used to construct a meta-matrix. Network analysis can be expressed in the form of a matrix or edge list, and the matrix was selected for effective expression of the properties and relationships found in the dataset, along with the weight of influence [20].

Weights are assigned through a one-to-one arrangement of decision-making factors in the LSPS, each indicating the weight of the influence that a source factor has on a target factor. As for the matrix construction process, the derived factors were arrayed against the entire data, and their degree of influence in each instance was reviewed by the panel of experts. Weights were subsequently assigned to these degrees of influence in the course of a further review. Correlations were found as shown in Table 6.

**Figure 4:**
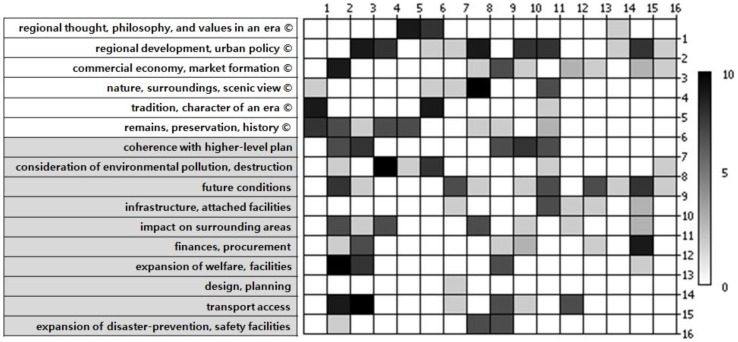
Matrix of technological and cultural factors.

In NetMiner 4.0, the matrix diagram shows the connections between factors. The cells in various shades (see Figure 4) at the intersections between factors with relative influence express the degree of influence between each factor in the matrix. Figure 4 below shows the analysis results of the techno-cultural factors’ degrees of influence.

### 3.3. Social Network Analysis

**Figure 5:**
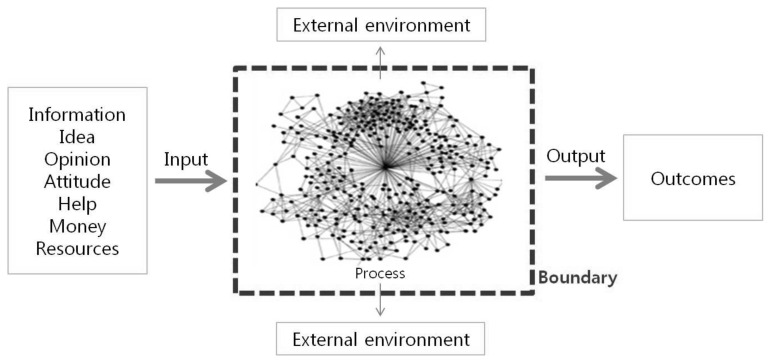
Network utilization in social network analysis.

Combining the words ‘social’ and ‘network’, [21], a social network signifies a network of interconnected persons or objects and represents an organic network formed through the interaction of diverse individuals [22]. Social network analysis thus determines the correlational structure of individuals or groups, focusing on how entities are connected and on identifying the nature of their connective structure [23], as shown in Figure 5.

As a method, social network analysis identifies and visualizes the centrality or importance of individual nodes by assigning weights to them [24]. These can subsequently be used to derive the various types of centrality—degree, closeness, betweenness, and eigenvector [25,26,27].

Degree centrality indicates the degree to which a node is connected to other nodes [28]. Closeness centrality expresses how close one node is to other nodes [29], and the higher the measure of this centrality, the easier it is for the node to be related to other nodes [30]. Betweenness centrality measures the degree to which one node acts as an intermediary in connecting other nodes into a network [31]. On the other hand, eigenvector centrality weighs the importance of nodes to obtain their centrality [32]. In a centrality analysis, the degree centrality is the basic measure, usually indicating the number of links connecting a given node to other nodes [33].

The equations below show how degree centrality and eigenvector centrality are used in social network analysis [34]:

(1) $$\begin{array}{ll} \mathrm{degree}\;\mathrm{centrality} & =\frac{\sum_{i=1}^{n}g_{i}{}^{\left(1\right)}}{n\left(n-1\right)}\\ & =\frac{\sum_{i=1}^{n}g_{i}{}^{\left(2\right)}}{n\left(n-1\right)},i=1,\ldots ,n \end{array}$$

Here $g_{i}{}^{\left(1\right)}$ is the number of inbound edges to the ith entity, and $g_{i}{}^{\left(2\right)}$ is the number of outbound edges from the ith entity.

The eigenvector centrality of node x is defined in terms of the following formula:

(2) $$\sigma_{E}(\chi )\;=\;v_{x}\;=\;\frac{1}{\lambda_{\max}\;(A)}\;\times \;\sum_{j=1}^{n}a_{jx}\times \;v_{j}$$

v = (v1, v2, vn) is the eigenvector for the maximum eigenvalue $\lambda_{\max}(A)$ of the entire matrix, and $a_{jx}$ represents the weight between node x and node j.

## 4. Results and Discussions: Correlation Analysis of Techno-Cultural Factors Affecting Policy Decision-Making through Social Network Analysis

Social network analysis of the decision-making factors in the LSPS was conducted using the constructed matrix. The 16 factors relevant to decision-making in the LSPS were analyzed in terms of degree centrality, eigenvector centrality, closeness centrality, betweenness centrality, and community structure. Visualizations of these analyses allowed for an intuitive grasp of the corresponding path models.

This study summarizes the results of social network analysis through the Appendix C, Appendix D, Appendix E and Appendix F (Table A2, Table A3, Table A4 and Table A5). Each Appendix’s table shows the effects of the 16 factors derived by ranking. Each numerical value in the table represents the effect between the factors. The degree of influence with other nodes was expressed numerically around individual factors according to the analysis theme (degree centrality, eigenvector centrality, closeness centrality, betweenness centrality, and community structure). Each indicator can be interpreted that the higher the number, the easier it is to relate to other nodes.

### 4.1. Degree Centrality Analysis

Degree centrality involves two separate measures: in-degree centrality indicating the degree to which an entity is influenced by other factors; and out-degree centrality indicating the degree to which an entity influences other factors. The present study derived each factor’s degree centrality by referring to the mean values of its in-degree and out-degree centrality.

Degree centrality is the most basic indicator of centrality in social network analysis that measures the importance of a given node in a network. A greater number of direct connections with other nodes determine a higher value of degree centrality. Previous studies used degree centrality to derive the importance of each node. However, this study cannot determine a node’s influence throughout the entire network through exclusive consideration of its direct connections. To overcome this limitation, eigenvector centrality was used.

Analysis of degree centrality for the technological and cultural factors corresponding to the classification codes given in Table 7 showed that the following had the highest values in terms of in-degree centrality: ‘regional development, urban policy’, followed by ‘future conditions’, ‘commercial economy, market formation’, and ‘impact on surrounding areas’.

**Figure 6:**
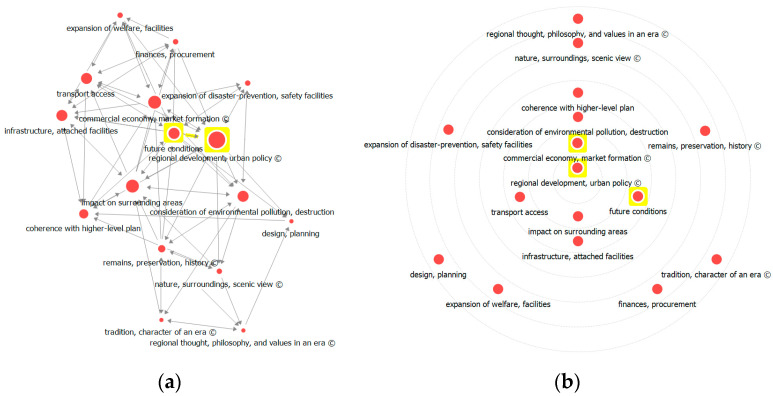
Visualization of degree centrality analysis: (**a**) network visualization and (**b**) circle diagram.

Regarding out-degree centrality, which measures the degree to which a factor influences other factors, ‘regional development, urban policy’, ‘future conditions’, ‘commercial economy, market formation’, and ‘impact on surrounding areas’ ranked higher, following the case of in-degree centrality. ‘Remains, preservation, history’ and ‘finances, procurement’ have relatively higher out-degree centrality than in-degree centrality. The higher values of out-degree and in-degree centralities indicate a greater number of nodes that are directly connected with one another, or a greater number of factors that influence and are influenced by other factors. Moreover, network visualization analysis (Figure 6) showed that ‘regional development, urban policy’ has the highest weight, followed by ‘future conditions’, ‘commercial economy, market formation’, ‘impact on surrounding area’, and so on. Circle diagram analysis locates the core factors with a greater centrality of influence nearer the center, and it was shown that ‘regional development, urban policy’ has the greatest centrality.

### 4.2. Eigenvector Centrality Analysis

The Eigenvector centrality analysis supplemented the degree centrality analysis results. Eigenvector centrality measures the centrality of a given node by weighing the importance of other nodes connected to it. Most of the factors with greater in-degree and out-degree centrality values—‘regional development, urban policy’, ‘future conditions’, ‘coherence with higher-level plan’, ‘impact on surrounding areas’, and ‘commercial economy, market formation’—also showed greater eigenvector centrality. Such results indicate that the said factors have more connections with other factors with great influence and, thus, relatively have a great influence on the entire network as well.

**Figure 7:**
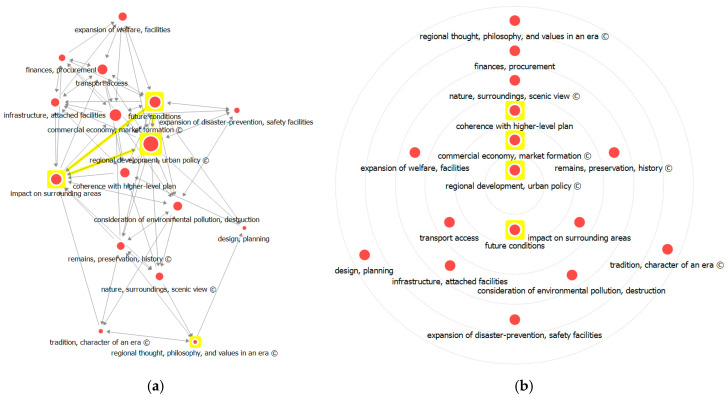
Visualization of eigenvector centrality analysis: (**a**) network visualization and (**b**) circle diagram.

Analysis results for both degree and eigenvector centralities indicate high values for ‘future conditions’ and economic factors as items in the policy-related consensus. Thus, we can see that these factors exert great influence on matters involving regional development and social change, which can be interpreted as highlighting the importance of planned regional development. Network visualization of the eigenvalue centrality analysis (Figure 7) also indicated that ‘regional development, urban policy’ has the highest weight, followed by ‘future conditions’, ‘coherence with higher-level plan’, and ‘impact on surrounding areas’. The circle diagram analysis locates the core factors with a greater centrality of influence nearer to the center; ‘regional development, urban policy’ showed the greatest centrality.

### 4.3. Closeness Centrality Analysis

Closeness centrality shows the immediacy of connections between factors: the shorter the distance between a given node and others, the greater is the relationship between them. This is because a shorter distance implies greater accessibility, thus allowing relatively quick access to other nodes through that pathway than others.

Analysis results for in-closeness centrality indicated high values for ‘regional development, urban policy’, ‘future conditions’, ‘commercial economy, market formation’, and ‘impact on surrounding areas’. These can be regarded as factors likely to be easily influenced by other factors. It is worth noting that ‘remains, preservation, history’ and ‘commercial economy, market formation in an area’ were shown to have relatively high centrality. As these are likely to be easily influenced by other factors, they may serve as important indicators for policy implications.

**Figure 8:**
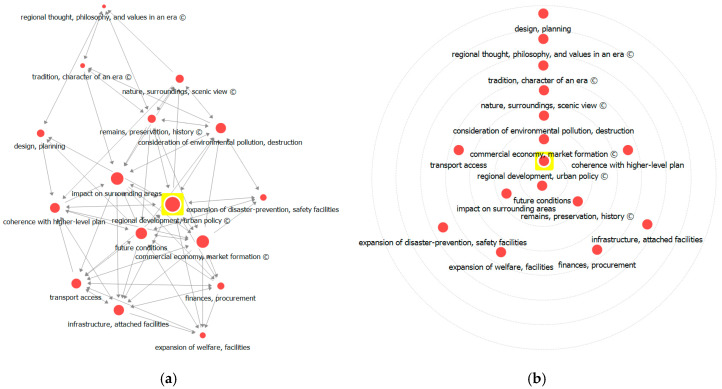
Visualization of closeness centrality analysis: (**a**) network visualization and (**b**) circle diagram.

Analysis results for out-closeness centrality (Figure 8) also indicated high values for ‘regional development, urban policy’, ‘future conditions’, ‘commercial economy, market formation’, and ‘impact on surrounding areas’. Note though that factors related to a region’s cultural connections and historical influence—‘remains, preservation, history’ and ‘regional thought, philosophy, and values in an era’—showed relatively high centrality. As these factors are also likely to influence other factors easily, they may serve as important indicators for social issues and implications related to decision-making in the LSPS.

Regarding the results of overall closeness centrality analysis, ‘regional development, urban policy’ showed the highest weight in decision-making during the LSPS, followed by ‘future conditions’ and ‘commercial economy, market formation’. Circle diagram analysis locates the core factors with greater centrality of influence nearer to the center; ‘regional development, urban policy’ was at the center, along with ‘coherence with higher-level plan’ and ‘commercial economy, market formation’ possessing high scores in terms of in-closeness and out-closeness centralities.

### 4.4. Betweenness Centrality Analysis

Betweenness centrality indicates a given factor’s intermediary role in connecting other factors. Factors frequently included in the shortest paths between other factors have high betweenness centrality, and they play a significant role in the network information flow. If such a factor is removed, it can have large repercussions on the overall flow and connectivity of the network. Betweenness centrality is thus an important measure of the intermediary role of factors.

Similar to the earlier results, analysis results for betweenness centrality indicated high values for ‘regional development, urban policy’, ‘future conditions’, and ‘impact on surrounding areas’. As highly ranked factors in betweenness centrality, these occupy an important place in regulating the overall flow among the 16 factors.

**Figure 9:**
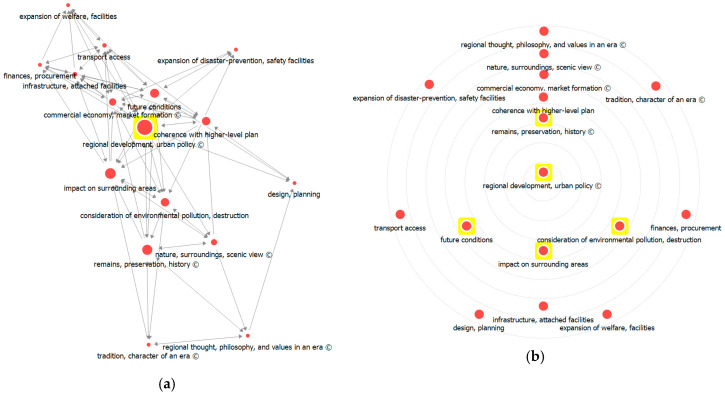
Visualization of betweenness centrality analysis: (**a**) network visualization and (**b**) circle diagram.

Overall analysis results for betweenness centrality (Figure 9) showed that ‘regional development, urban policy’ has the highest weight, followed by ‘future conditions’ and ‘impact on surrounding areas’. Circle diagram analysis locates the core factors with the greater centrality of influence nearer to the center. The yellow highlight in the diagram (see Figure 9) indicates that ‘regional development, urban policy’ has far greater centrality than the other factors.

### 4.5. Community Structure Analysis

Community structure analysis facilitates the identification of a network structure that comprises different cohesive groups of closely connected nodes. This enables the understanding of the connective properties of a network through the characteristics of cohesive groups and the relationships obtained among them. Factors were sorted into different groups; the assigned numbers are for labeling purposes and does not indicate their ranking [35].

**Figure 10:**
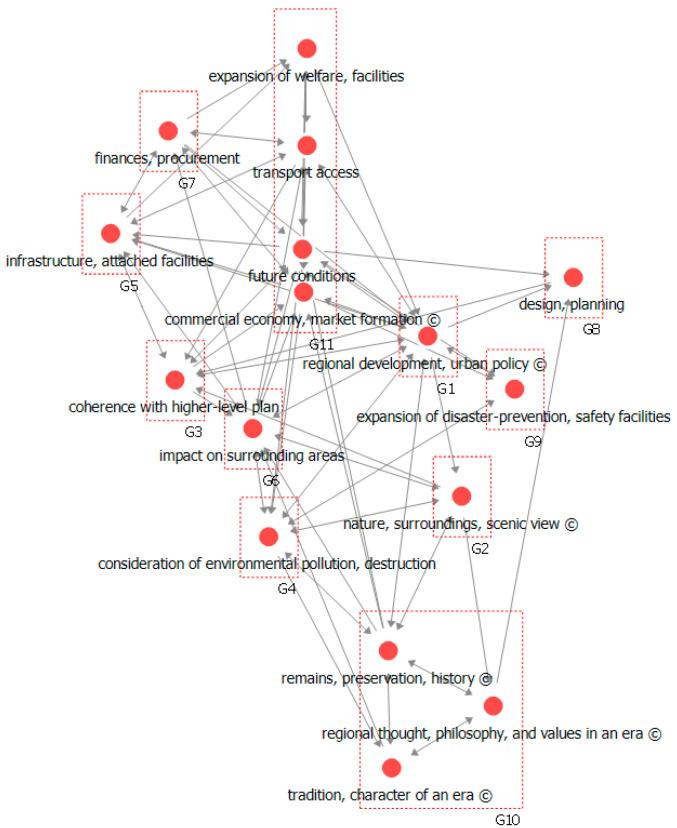
Visualization of community structure analysis.

As shown in Table 8 and Figure 10, the results of the community structure analysis indicate that ‘regional development, urban policy’, was shown to have high values in centrality metrics, belonging to a separate group. ‘Future conditions’, ‘expansion of welfare facilities’, ‘transport access’, and ‘commercial economy, market formation’ adhere together in another group. In particular, three cultural factors, ‘regional thought, philosophy, values in an era’, ‘tradition, character of an era’, and ‘remains, preservation, history’ adhered together in a separate group. When overall relationships among the factors are considered, it was found that the important cultural factors had somewhat less centrality of influence on the main technological factors.

Note that groups of factors identified as important in the earlier centrality analysis, such as G1 (regional development, urban policy), G3 (coherence with higher-level plan), G4 (consideration of environmental pollution, destruction), and G6 (impact on surrounding areas), play a central role in the relationships among factors, albeit not as functional factors but as various convergent factors involved in the decision-making of the LSPS. They also constitute independent entities to which core meanings can be assigned.

The community structure analysis results show that various factors in the classification code have even influence on cultural influence factors. Understanding the decision-making in the LSPS is not only the visible ‘technological factors’ such as structure, form, and construction method but also the invisible ‘cultural factors’ such as the way of life projected in the process of space creation, the spirit of the times, religion and scholarship including intellectual insight into the arts.

In terms of social network analysis and utilization of big data conducted in this study, the use of various data-focused on local space is gradually increasing due to the development of information and communication technology for the change of local spaces and policy decision making scheduled for development. In other words, traditional urban spaces are being developed conceptually from intelligent city to smart city, with automation and the development of land communication technology. Additionally, with this trend, the local space is developing smarter by using various information. In particular, in terms of the characteristics of convergence space data utilization in this study, various spatial information is used in planning and design coordination through social trend monitoring. Such information analysis can be used as data for policy decision-making, such as conflict management in the community and gathering opinions on public policies. In the future, big data analysis, including social network analysis and text mining analysis, is expected to be highly valuable as a methodology for regional development and spatial planning policies.

The convergent influencing factor of local construction policy is the element that comprehensively develops the elements of human lifestyle or phenomenal society, including industrial production and consumption activities from a technological and cultural point of view. This implies emotional factors that can lead to practical communication and empathy to realize cultural-heritage value creation, tourism resource development, and storytelling for the community.

Therefore, it is the product of the local space where the cultural value at the LSPS cannot be just explained as simple morphological and tangible information. Various studies that would deeply examine the existence of various intangible factors surrounding the space with the formation of technical space are thus necessary.

In another aspect, this study expects that it will be a policy material that can be used selectively in policy decision-making not only in Korea but also in areas similar to Korea. The information in this study is expected to be useful, especially as a result of cultural factors that cannot be ignored for regional development. In order to enhance this utilization, we will conduct future research by establishing an area that can be applied to the case based on the results obtained.

However, the applied methodology has limitations in comprehensively analyzing local policy decisions. There is also a limit to the application methodology to determine the local applicability of the analyzed cultural factors. In order to enhance this utilization, it is necessary to select areas that can be applied to the analyzed cases based on the results obtained and to conduct future research related to these verifications.

## 5. Conclusions

This study’s approach derived more objective information from the data using text mining and social network analyses. To increase the objective reliability of the data, the Delphi method was used for a correlational analysis of the factors that can influence policy decision-making in the spatial planning stage.

Prior to the social network analysis, the Delphi technique and text mining data analytics were utilized to collect and investigate a wide range of convergent data. We ran a group of experts three times through the Delphi technique. We surveyed and interviewed a group of experts three times using the Delphi technique. A total of 31 experts in seven fields, including construction environment and spatial culture, were used to derive key indicators of techno-cultural factors. Social network analysis was then used to identify the connections between the factors relevant to decision-making in the LSPS from a convergent perspective, consequently determining the influence of these factors.

Through text mining analysis, 16 key factors that affect decision-making in the spatial planning stage were derived. The keyword found with the most influential factor was under the regional development and urban policy category, which is related to various local construction ideas based on: basic social issues concerning regional development such as policy agenda; mid- to long-term urban planning; and collection of various opinions for local development and policy-making. This factor is directly connected to various factors, exerting high influence, as well as being highly influenced by, other factors.

Looking at the cultural aspects of the 16 factors from a convergent perspective, ‘commercial economy, market formation’ and ‘remains, preservation, history’ were found to have a higher influence than expected. This showed that highly ranked factors are important in regulating the overall flow among the 16 factors. In particular, ‘remains, preservation, history’ has a higher incoming influence than outgoing influence; it is expected that this will have a relative impact on regional development. Therefore, decision-making in regard to regional development should be re-examined to ensure that a socially conventional approach to cultural properties and historical sites in regional development is not confined to historical preservation. It was found that ‘regional thought, philosophy, and values in an era’ also has a relatively high level of incoming influence. Thus, it is a factor that can be likely influenced by other factors and can serve as a key indicator of policy decision-making.

Among the factors subjected to community structure analysis, ‘regional development, urban policy’, which exhibited high values in centrality metrics, was found to belong to a separate group, and ‘future conditions’, ‘expansion of welfare facilities’, ‘transport access’, and ‘commercial economy, market formation’ adhered together in another group. In particular, two cultural factors, namely, ‘regional thought, philosophy, values in an era’ and ‘tradition, character of an era’, adhered together in a separate group. When considering the overall relationships among the factors, it was found that the important cultural factors had somewhat less centrality of influence on the main technological factors.

It was found that the cultural factors ‘regional thought, philosophy, values in an era’ and ‘tradition, character of an era’ had little direct influence on ‘regional development, urban policy’, which was identified as the main influential factor. Such a phenomenon may be interpreted as indicating that cultural keywords or factors do not directly influence the core of construction policy. Such lack of cultural contents can inhibit regional growth; policy supplementation should thus be included. For a balanced expression of the diversity and uniqueness of each region, as the basis of regional development, convergent policies need to be utilized and strategic local policies that can actively promote industrial growth, tourism and local branding with an emphasis on bringing out the overall, traditional characteristics of each region should be implemented.

## Figures and Tables

**Table 1:** Collected Research Papers.

Search Category	Number of Academic Papers	Total No. of Extracted Words
Construction + Culture + Region	471	78,112
Civilworks + Culture + Region	34	4490
National land + Culture + Region	239	34,778
City + Culture + Region	961	112,166

**Table 2:** Top 30 Repeated Keywords.

Search Category (Papers)	Top 30 Repeated Keywords
Construction + Culture + Region (471 papers)	region, society, culture, construction, city, economy, problem, growth, relationship, state, nation, policy, history, persons with disabilities, politics, development, space, change, education, China, government, tradition, unification, promotion, diversity, world, strategy, security, the people, citizens
Civil works + Culture + Region (34 papers)	region, culture, informatization, scenic view, change, development, space, railroad, history, visualization, heritage, construction, bridge, place name, ground, travel, nature, characteristics, distinctive character, building, technology, building information modeling, value, element, location, computer, civil works, economy, ancient burial mound, planning
National Land + Culture + Region (239 papers)	culture, development, region, economy, society, environment, city, growth, scenic view, state, government, innovation, national land, relationship, corporation, analysis, cluster, world, space, technology, policy, change, assessment, the people, project, region, tourism, management, plan, industry
City + Culture + Region (961 papers)	city, region, culture, space, society, design, development, environment, history, tradition, diversity, economy, town, growth, scenic view, change, industry, public, identity, purpose, woman, policy, project, value, place, life, residents, festival, Busan, creation

**Table 3:** Delphi Research Design.

Stage		Content							
Preliminary basic research	Task	Examining keywords and data for techno-cultural factor analysis							
	Experts	Field of expertise	Construction				Spatial		Regional
			Environment	Work	Design	Management	Culture	Philosophy	Policy
		Number of participants	1	1		2	1	1	
Delphi research	Task	Examining the derivation of indicators for and assignment of weights to techno-cultural factors for social network analysis							
	Round 1	Delphi panel selection Classification of data and examination of indicators Open-ended questionnaire, interview, e-mail questionnaire	1	1	3	2	1	2	1
	Round 2	Supplementation of derived indicators from round 1 Open-ended questionnaire, interview, email questionnaire	1	2	1	2	1	1	2
	Round 3	Review of factor indicators and assignment of weights for data accuracy Open-ended questionnaire, interview, e-mail questionnaire	1	2	1	3	1	1	1

**Table 4:** Indicators derived through the Delphi Survey.

Round	Survey Content	Derived Indicators			Participating Experts
		Technological Factors	Cultural Factors	Techno-Cultural	
1st	Examination of factor indicators	30	45	17	11
2nd	Supplementation of factor indicators	18	33	17	10
3rd	Review of factor indicators/weight assignment	16	31	16	10

**Table 5:** Factor Indicators derived through three Rounds of Delphi Survey.

Technological Influence	Cultural Influence		Techno-Cultural Factors
regional thought, philosophy, and values in an era	region	construction	coherence with related plans
regional development, urban policy	culture	China	local environment
commercial economy, market formation	society	industry	natural environment
nature, surroundings, scenic view	space	globalization	future conditions
tradition, the character of an era	development	project feasibility	resident environment
remains, preservation, history	economy	strategy	infrastructure
coherence with higher-level plan	growth	value	impact on surrounding areas
consideration of environmental pollution, destruction	history	scenic view	site scale
future conditions	environment	government	finances, procurement
infrastructure, attached facilities	change	identity	public contribution
impact on surrounding areas	policy	town	suitability of development density
finances, procurement	design	education	placement of facilities
expansion of welfare, facilities	characteristics	residents	scenic view plan
design, planning	diversity	plan	environmental pollution
transport access	state	social perception	transport convenience
expansion of disaster-prevention, safety facilities	tradition		safety facilities

**Table 6:** Correlations between Factors and Degree of Influence.

	B ^1^	Regional Thought, Philosophy, and Values in an Era ©	Regional Development, Urban Policy ©	Commercial Economy, Market Formation ©	Nature, Surroundings, Scenic View ©	Tradition, Character of an Era ©	Remains, Preservation, History ©	Coherence with Higher-Level Plan	Consideration of Environmental Pollution Destruction	Future Conditions	Infrastructure, Attached Facilities	Impact on Surrounding Areas	Finances, Procurement	Expansion of Welfare, Facilities	Design, Planning	Transport Access	Expansion of Disaster-Prevention, Safety Facilities
A ^1^																	
regional thought, philosophy, and values in an era ©		0.000	3.429	3.571	2.714	7.286	6.429	2.571	0.857	4.143	2.143	3.571	1.286	1.714	4.143	1.286	1.000
regional development, urban policy ©		2.714	0.000	6.857	5.857	2.143	4.571	4.143	6.429	4.000	6.429	6.571	3.000	3.714	4.143	6.286	4.000
commercial economy, market formation ©		2.286	6.714	0.000	2.857	2.143	1.571	2.571	3.429	4.857	4.571	3.143	5.143	3.714	4.000	5.286	4.429
nature, surroundings, scenic view ©		3.429	3.429	2.429	0.000	2.571	3.714	3.000	6.857	3.714	2.143	5.286	1.143	1.000	3.571	2.714	1.000
tradition, character of an era ©		7.143	3.143	2.857	2.429	0.000	6.714	1.571	2.429	2.143	1.571	3.571	1.000	1.143	2.429	1.714	1.000
remains, preservation, history ©		5.857	5.000	3.857	4.857	5.000	0.000	3.143	4.571	4.286	2.571	5.000	2.000	1.857	3.000	3.714	2.286
coherence with higher-level plan		1.000	5.714	6.286	3.429	3.000	3.429	0.000	3.571	5.429	6.429	5.143	4.000	3.714	3.429	3.714	3.143
consideration of environmental pollution, destruction		2.286	3.571	1.429	8.000	3.000	6.429	2.571	0.000	3.000	2.571	3.429	2.286	0.714	3.286	1.000	3.571
future conditions		2.714	6.429	4.857	3.000	0.857	3.571	3.857	3.286	0.000	4.143	5.429	3.857	5.429	4.571	5.857	4.000
infrastructure, attached facilities		1.429	3.571	2.571	1.714	0.857	0.857	4.429	2.714	3.143	0.000	5.143	3.000	4.143	2.857	5.000	2.714
impact on surrounding areas		1.143	4.857	4.143	4.571	1.857	2.286	2.714	4.000	3.429	4.000	0.000	3.857	2.429	2.857	4.000	1.143
finances, procurement		1.286	4.143	5.143	1.857	0.857	2.000	4.000	1.429	4.286	3.857	2.571	0.000	4.286	3.714	6.714	3.143
expansion of welfare, facilities		1.857	6.857	4.714	1.000	0.000	0.000	2.857	0.286	4.857	2.857	3.429	1.429	0.000	2.000	3.143	3.000
design, planning		2.857	2.857	3.143	4.000	2.286	3.143	3.143	3.143	3.857	2.571	3.143	1.714	1.143	0.000	3.000	3.000
transport access		0.000	6.857	8.000	2.286	0.429	1.143	3.000	0.000	5.000	4.429	3.857	4.571	0.000	1.000	0.000	0.714
expansion of disaster-prevention, safety facilities		0.000	3.143	2.857	1.571	0.000	1.143	2.857	3.857	5.000	3.143	0.857	0.000	0.714	3.143	0.000	0.000

^1^ Directionality → (A → B, the strength and direction of an association between A and B).

**Table 7:** Classification of Cultural and Technological Factors: Round 1 of the Delphi Consultation.

Classification Codes		Associated Keywords
Cultural factors	ethos, thought, religion	regional thought, philosophy, and values in an era
	plan, system, policy	regional development, urban policy
	society, economy	commercial economy, market formation
	geography, environment	nature, surroundings, scenic view
	tradition, custom	tradition, character of an era
	culture, inheritance	remains, preservation, history
Technological factors	connection with other plans	coherence with higher-level plan
	environment	consideration of environmental pollution, destruction
	growth potential	future conditions
	amenities plan	infrastructure, attached facilities
	site scale, location	impact on surrounding areas
	development costs	finances, procurement
	publicness	expansion of welfare, facilities
	scenic view	design, planning
	transportation	transport access
	safety	expansion of disaster-prevention, safety facilities

**Table 8:** Community Structure Analysis Results.

Community	Included Factors ※(C): Cultural Factor
G1	regional development, urban policy (C)
G2	nature, surroundings, scenic view (C)
G3	coherence with higher-level plan
G4	consideration of environmental pollution, destruction
G5	infrastructure, attached facilities
G6	impact on surrounding areas
G7	finances, procurement
G8	design, planning
G9	expansion of disaster-prevention, safety facilities
G10	remains, preservation, history (C) + regional thought, philosophy, and values in an era (C) + tradition, character of an era (C)
G11	expansion of welfare, facilities + transport access + future conditions + commercial economy, market formation (C)

**Figure A1:**
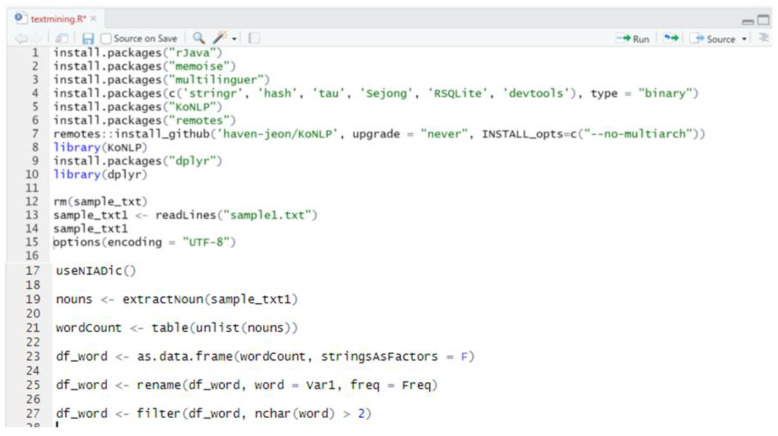
Source Code for Text mining.

**Figure A2:**
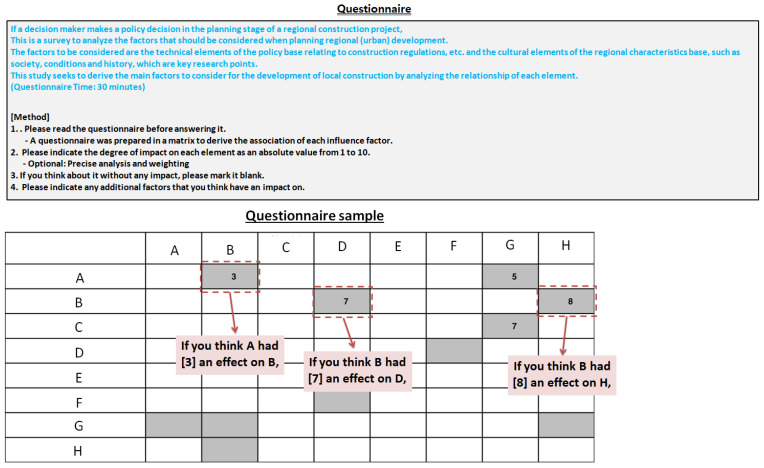
Questionnaires introduction page.

**Figure A3:**
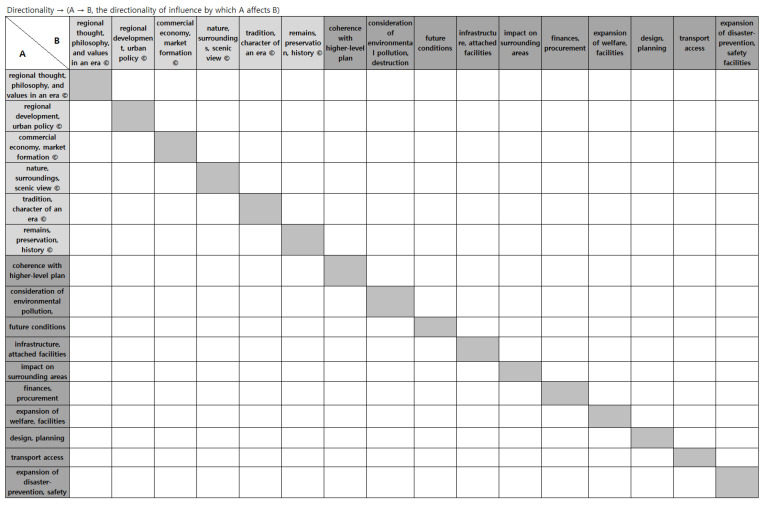
Questionnaires sample for Delphi research.

## Appendix A

**Table A1:** Keyword frequency in the Target Research Data Set.

Rank	Keyword	Frequency	Rank	Keyword	Frequency
1	region	2827	26	identity	423
2	culture	2609	27	town	422
3	society	1516	28	education	421
4	space	1092	29	residents	420
5	development	1033	30	plan	405
6	economy	885	31	perception	403
7	growth	782	32	function	392
8	history	733	33	citizens	388
9	environment	697	34	life	380
10	change	665	35	policy measures	367
11	policy	663	36	region	367
12	design	654	37	tourism	354
13	characteristics	636	38	international	354
14	diversity	635	39	cultural property	354
15	state	615	40	nation	351
16	tradition	599	41	facilities	351
17	construction	587	42	place	349
18	China	553	43	politics	347
19	industry	527	44	technological support	345
20	world	490	45	technology	338
21	project	476	46	corporation	334
22	strategy	475	47	institutions	325
23	value	461	48	era	316
24	scenic view	457	49	promotion direction	315
25	government	450	50	system	315

## Appendix C

**Table A2:** In-Degree and out-Degree Centrality Results of Degree Centrality Analysis.

Factors ※(C): Cultural Factor	In-Degree Centrality	Rank	Factors ※(C): Cultural Factor	Out-Degree Centrality	Rank
regional development, urban policy (C)	0.667	1	regional development, urban policy (C)	0.733	1
future conditions	0.600	2	future conditions	0.667	2
commercial economy, market formation (C)	0.533	3	commercial economy, market formation (C)	0.600	3
impact on surrounding areas	0.533	3	remains, preservation, history (C)	0.533	4
coherence with higher-level plan	0.467	5	impact on surrounding areas	0.467	5
consideration of environmental pollution, destruction	0.467	5	finances, procurement	0.467	5
infrastructure, attached facilities	0.467	5	coherence with higher-level plan	0.400	7
transport access	0.467	5	consideration of environmental pollution, destruction	0.400	7
nature, surroundings, scenic view (C)	0.333	9	transport access	0.400	7
remains, preservation, history(C)	0.333	9	infrastructure, attached facilities	0.333	10
finances, procurement	0.333	9	nature, surroundings, scenic view (C)	0.333	10
expansion of welfare, facilities	0.267	12	expansion of welfare, facilities	0.267	12
design, planning	0.267	12	regional thought, philosophy, and values in an era (C)	0.267	12
expansion of disaster-prevention, safety facilities	0.267	12	expansion of disaster-prevention, safety facilities	0.200	14
regional thought, philosophy, and values in an era (C)	0.200	15	tradition, character of an era (C)	0.200	14
tradition, character of an era (C)	0.200	15	design, planning	0.133	16

## Appendix D

**Table A3:** Eigenvector Centrality Analysis Results.

Factors ※(C): Cultural Factor	Eigenvector Centrality	Rank
regional development, urban policy (C)	0.48370	1
future conditions	0.32974	2
coherence with higher-level plan	0.32551	3
impact on surrounding areas	0.31794	4
commercial economy, market formation (C)	0.31218	5
transport access	0.30071	6
infrastructure, attached facilities	0.25420	7
consideration of environmental pollution, destruction	0.22437	8
nature, surroundings, scenic view (C)	0.21272	9
expansion of welfare, facilities	0.19055	10
finances, procurement	0.17505	11
remains, preservation, history	0.13545	12
expansion of disaster-prevention, safety facilities	0.08194	13
tradition, character of an era (C)	0.06341	14
regional thought, philosophy, and values in an era (C)	0.05853	15
design, planning	0.05202	16

## Appendix E

**Table A4:** In-Closeness and out-Closeness Centrality Results of Closeness Centrality Analysis.

Factors ※(C): Cultural Factor	In-Closeness Centrality	Rank	Factors ※(C): Cultural Factor	Out-Closeness Centrality	Rank
regional development, urban policy (C)	0.750	1	regional development, urban policy (C)	0.789	1
future conditions	0.714	2	future conditions	0.714	2
commercial economy, market formation (C)	0.682	3	commercial economy, market formation (C)	0.682	3
impact on surrounding areas	0.682	3	remains, preservation, history (C)	0.682	4
consideration of environmental pollution, destruction	0.652	5	impact on surrounding areas	0.652	5
infrastructure, attached facilities	0.652	5	consideration of environmental pollution, destruction	0.600	6
coherence with higher-level plan	0.625	5	finances, procurement	0.600	6
transport access	0.625	5	coherence with higher-level plan	0.577	8
nature, surroundings, scenic view (C)	0.600	9	transport access	0.577	8
remains, preservation, history (C)	0.577	10	nature, surroundings, scenic view (C)	0.577	8
finances, procurement	0.556	11	regional thought, philosophy, and values in an era (C)	0.556	11
design, planning	0.556	11	expansion of welfare, facilities	0.536	12
expansion of disaster-prevention, safety facilities	0.517	13	expansion of disaster-prevention, safety facilities	0.517	13
expansion of welfare, facilities	0.500	14	infrastructure, attached facilities	0.500	14
tradition, character of an era (C)	0.455	15	tradition, character of an era (C)	0.500	14
regional thought, philosophy, and values in an era (C)	0.429	16	design, planning	0.469	16

## Appendix F

**Table A5:** Betweenness Centrality Analysis Results.

Factors ※(C): Cultural Factor	Betweenness Centrality	Rank
regional development, urban policy (C)	0.165	1
future conditions	0.112	2
impact on surrounding areas	0.101	3
consideration of environmental pollution, destruction	0.080	4
remains, preservation, history (C)	0.067	5
commercial economy, market formation (C)	0.064	6
nature, surroundings, scenic view (C)	0.062	7
coherence with higher-level plan	0.062	7
regional thought, philosophy, and values in an era (C)	0.021	9
infrastructure, attached facilities	0.020	10
finances, procurement	0.018	11
transport access	0.018	11
design, planning	0.011	13
tradition, character of an era (C)	0.006	14
expansion of welfare, facilities	0.006	14
expansion of disaster-prevention, safety facilities	0.002	16
